# Determination of the melon chloroplast and mitochondrial genome sequences reveals that the largest reported mitochondrial genome in plants contains a significant amount of DNA having a nuclear origin

**DOI:** 10.1186/1471-2164-12-424

**Published:** 2011-08-20

**Authors:** Luis Rodríguez-Moreno, Víctor M González, Andrej Benjak, M Carmen Martí, Pere Puigdomènech, Miguel A Aranda, Jordi Garcia-Mas

**Affiliations:** 1 Departamento de Biología del Estrés y Patología Vegetal, Centro de Edafología y Biología Aplicada del Segura (CEBAS)-CSIC, 30100 Espinardo (Murcia), Spain; 2 Department of Molecular Genetics, Centre for Research in Agricultural Genomics CSIC-IRTA-UAB, UAB Campus, Edifici CRAG, 08193 Bellaterra (Barcelona), Spain; 3 IRTA, Centre for Research in Agricultural Genomics CSIC-IRTA-UAB, Campus UAB, Edifici CRAG, 08193 Bellaterra, (Barcelona), Spain

## Abstract

**Background:**

The melon belongs to the *Cucurbitaceae *family, whose economic importance among vegetable crops is second only to *Solanaceae*. The melon has a small genome size (454 Mb), which makes it suitable for molecular and genetic studies. Despite similar nuclear and chloroplast genome sizes, cucurbits show great variation when their mitochondrial genomes are compared. The melon possesses the largest plant mitochondrial genome, as much as eight times larger than that of other cucurbits.

**Results:**

The nucleotide sequences of the melon chloroplast and mitochondrial genomes were determined. The chloroplast genome (156,017 bp) included 132 genes, with 98 single-copy genes dispersed between the small (SSC) and large (LSC) single-copy regions and 17 duplicated genes in the inverted repeat regions (IRa and IRb). A comparison of the cucumber and melon chloroplast genomes showed differences in only approximately 5% of nucleotides, mainly due to short indels and SNPs. Additionally, 2.74 Mb of mitochondrial sequence, accounting for 95% of the estimated mitochondrial genome size, were assembled into five scaffolds and four additional unscaffolded contigs. An 84% of the mitochondrial genome is contained in a single scaffold. The gene-coding region accounted for 1.7% (45,926 bp) of the total sequence, including 51 protein-coding genes, 4 conserved ORFs, 3 rRNA genes and 24 tRNA genes. Despite the differences observed in the mitochondrial genome sizes of cucurbit species, *Citrullus lanatus *(379 kb), *Cucurbita pepo *(983 kb) and *Cucumis melo *(2,740 kb) share 120 kb of sequence, including the predicted protein-coding regions. Nevertheless, melon contained a high number of repetitive sequences and a high content of DNA of nuclear origin, which represented 42% and 47% of the total sequence, respectively.

**Conclusions:**

Whereas the size and gene organisation of chloroplast genomes are similar among the cucurbit species, mitochondrial genomes show a wide variety of sizes, with a non-conserved structure both in gene number and organisation, as well as in the features of the noncoding DNA. The transfer of nuclear DNA to the melon mitochondrial genome and the high proportion of repetitive DNA appear to explain the size of the largest mitochondrial genome reported so far.

## Background

The melon (*Cucumis melo *L.) is an important vegetable crop grown in temperate, subtropical and tropical regions worldwide. The melon belongs to the *Cucurbitaceae *family, which also comprises other vegetable crops such as cucumber, watermelon, pumpkin and squash, and whose economic importance among vegetable crops is second only to *Solanaceae*. *C. melo *is a diploid species (2x = 2n = 24) with an estimated haploid genome size of 454 Mb [1]. In recent years, extensive research has been performed in melon to elucidate fruit ripening processes, carotene accumulation and aroma production [2]. In addition, genomic approaches to melon breeding have been successfully applied to the molecular characterisation of important agronomic traits, such as pathogen resistance [3,4] and sex determination [5,6]. Recent research has increased the availability of genetic and genomic resources for melon [7], such as the sequencing of ESTs [8,9], the development of an oligonucleotide-based microarray [10], the construction of BAC libraries [11-13], the production of mutant collections for TILLING analyses [14-16], the development of a collection of near-isogenic lines (NILs) [17], the construction of several genetic maps [9,18-22] and the development of a genetically anchored BAC-based physical map [23].

The MELONOMICS project, aimed at sequencing the complete melon genome using a whole-genome shotgun strategy, was recently initiated by a Spanish consortium [24]. Determination of the complete melon genome also includes sequencing of the chloroplast (cpDNA) and mitochondrial (mtDNA) genomes. As of 6 June 2011, the NCBI databases contain 220 *eukaryota *plastid genome records [25]. Comparative studies have indicated that the chloroplast genomes of land plants are highly conserved in both gene order and gene content and are moderately sized, between 130 and 150 kb [26]. In contrast, plant mitochondrial genomes range from 200 to 2,400 kb in size, which is at least 10 to 100 times the size of typical animal mitochondrial genomes [27,28]. *Cucurbitaceae *possess the largest known plant mitochondrial genomes; however, species that belong to the same genera within *Cucurbitaceae *and have similar nuclear genome sizes show great size differences in their mitochondrial genomes [27]. Experimental procedures based on kinetic reassociation rate measurements have predicted a melon mitochondrial genome of 2,400 kb, the largest one among plants and animals and comparable in size to the genomes of many free-living bacteria [27,29]. Recently, the mitochondrial genomes of *Citrullus lanatus *(watermelon) (379 kb) and *Cucurbita pepo *(squash) (983 kb) have been determined [30]. Sequence analysis of these mitochondrial DNAs has suggested that the increased genome size in this family reflects an accumulation of chloroplast-derived and short repeated sequences, whereas protein-coding regions are conserved across these species, with minor exceptions [30,31]. In general terms, DNA transfer from organellar genomes to nuclear DNA, and *vice versa*, appears to be a common phenomenon associated with the redistribution of genetic material between nuclear and organellar genomes [32-35]. Furthermore, a reduction in organelle DNA content is linked to a gradual loss of the genetic autonomy of organelles [34,36,37].

Next-generation sequencing platforms are rapidly changing the field of genomics, allowing both re-sequencing and *de novo *sequencing of whole genomes with a significant reduction in cost and time relative to conventional approaches. Nevertheless, only a few examples of plastid genome next-generation sequencing have been published so far and no plant mitochondrial genome has been sequenced that way [38-44]. In this article, we report the complete sequence of the melon chloroplast genome obtained from BAC end sequences (BES), and we report an estimated 95% of the melon mitochondrial genome determined using Roche-454 sequencing technology. With a size over 2.7 Mb, the mitochondrial genome of melon represents the largest mitochondrial genome sequenced so far. Data on the structure and content of both organellar genomes and a comparison to published cucumber chloroplast and watermelon and squash mitochondrial genomes are presented.

## Results and discussion

### Organisation of the *Cucumis melo *chloroplast genome

**Figure 1:**
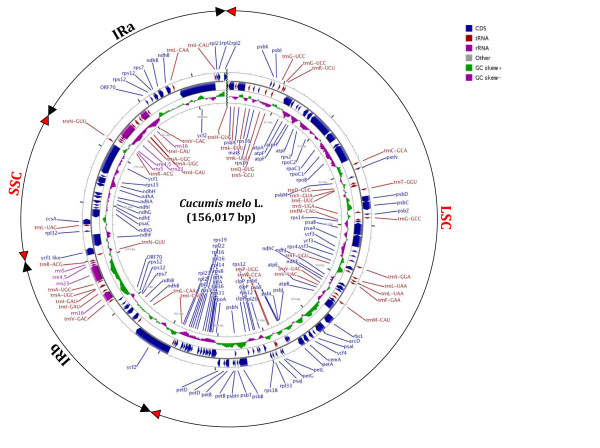
**Gene map of the *Cucumis melo *chloroplast genome**. The nucleotide positions are numbered starting at the IRa/LSC junction and extending clockwise. A pair of inverted repeats, IRb and IRa, located at coordinates 86,335 to 112,131 and 130,221 to 156,017, respectively, separates the large single-copy region (LSC) from the small single-copy region (SSC).

The complete nucleotide sequence of the chloroplast genome of melon (*C. melo *subsp. *melo*, PIT92) was determined (GenBank Acc. No. JF412791). The genome was 156,017 bp long and included a pair of inverted repeats (IRa and IRb) of 25,797 bp separated by small (SSC) and large (LSC) single-copy regions of 18,090 and 86,334 bp, respectively (Figure 1, Table 1). The GC content was found to be 36.9%, which is identical to that of cucumber, the only other reported cucurbit chloroplast genome [45-47], and to other sequenced plant chloroplast genomes.

**Table 1:** *C. melo *chloroplast genome characteristics

**Total size [nt]**	156,017
**GC content**	36.9%
**Gene number**	132^a^
**Protein genes**	87 (81)^b^
**rRNA genes**	8 (4)^b^
**tRNA genes**	37 (30)^b^
**Single-copy genes**	98
**Duplicated genes**	17
**Gene with introns**	18
**Trans-spliced genes**	1
**Coding sequences [nt]**	93,209 (59.7%)
**Protein coding [nt]**	80,580 (51.6%)
**tRNAs and rRNAs [nt]**	12,629 (8.1%)
**Non-coding sequences [nt]**	62,809 (40.3%)
***cis*-spliced introns [nt]**	18,822 (12.1%)
**Intergenic sequences [nt]**	43,987 (28.2%)

^a^Duplicated genes counted as two

^b^In parentheses, duplicated genes counted as one

The melon chloroplast genome contains 132 genes, including 98 single-copy genes and 17 duplicated in IR regions (Figure 1 and Table 2). The gene-coding regions accounted for 59.7% of the genome and included 75 protein-coding genes and 6 conserved ORFs, 4 rRNA genes and 30 tRNA genes, which represented 51.6%, 2.9% and 5.2% of the total sequence, respectively; *cis*-spliced introns accounted for 12.1% of the genome. The genes *clpP, rps12 and ycf3 *contained two introns, while 15 additional genes contained one intron each. The *rps12 *gene was found to undergo *trans*-splicing, with the 5' exon located in the LSC region and the other two exons located in both IR regions.

**Table 2:** List of genes found in the *Cucumis melo *chloroplast genome

**RNA genes**					
**tRNAs**	*trnA*-UGC^a, b^	*trnC*-GCA	*trnD*-GUC	*trn*E-UUC	*trn*F-GAA
	*trnfM*-CAU	*trnG*-GCC	*trnG*-UCC^a^	*trn*H-GUG	*trn*I-CAU^b^
	*trnI*-GAU^a, b^	*trnK*-UUU^a^	*trnL*-CAA^b^	*trn*L-UAA^a^	*trn*L-UAG
	*trnM*-CAU	*trnN*-GUU^b^	*trnP*-UGG	*trn*Q-UUG	*trn*R-ACG^b^
	*trnR*-UCU	*trnS*-GCU	*trnS*-GGA	*trn*S-UGA	*trn*T-GGU
	*trnT*-UGU	*trnV*-GAC^b^	*trnV*-UAC^a^	*trn*W-CCA	*trn*Y-GUA
**rRNAs**	*rrn16*^b^	*rrn23*^b^	*rrn4.5*^b^	*rrn*5^b^	
**Photosynthesis genes**					
**Acetyl-coa carboxylase**	*accD*				
**ATP-dependent protease**	*clpP*^c^				
**ATP synthase**	*atpA*	*atpB*	*atpE*	*atpF*^a^	*atpH*
	*atpI*				
**Cytochrome *b*/*f***	*petA*	*petB*^a^	*petD*^a^	*petG*	*petL*
	*petN*				
**Cytochrome *c *biogenesis**	*ccsA*				
**NADH dehydrogenase**	*ndhA*^a^	*ndhB*^a, b^	*ndhC*	*ndhD*	*ndhE*
	*ndhF*	*ndhG*	*ndhH*	*ndhI*	*ndhJ*
	*ndhK*				
**Photosystem I**	*psaA*	*psaB*	*psaC*	*psaI*	*psaJ*
**Photosystem II**	*psbA*	*psbB*	*psbC*	*psbD*	*psbE*
	*psbF*	*psbH*	*psbI*	*psbJ*	*psbK*
	*psbL*	*psbM*	*psbN*	*psbT*	*psbZ*
**Rubisco**	*rbcL*				
**Other genes**					
**Conserved ORFs**	*ycf1*	*ycf1*-like^d^	*ycf2*^b^	*ycf3*^c^	*ycf4*
	ORF70^b, e^				
**Transl. initiation factor**	*infA*				
**Intron maturase**	*matK*				
**Membrane protein**	*cemA*				
**Ribosomal proteins**	*rpl14*	*rpl16*^a^	*rpl2*^a, b^	*rpl20*	*rpl22*
	*rpl23*^b^	*rpl32*	*rpl33*	*rpl36*	*rps2*
	*rps3*	*rps4*	*rps7*^b^	*rps8*	*rps11*
	*rps12*^b, c, f^	*rps14*	*rps15*	*rps16*^a^	*rps18*
	*rps19*				
**RNA polymerase**	*rpoA*	*rpoB*	*rpoC1*^a^	*rpoC2*	

^a^Gene that contains one intron

^b^Two gene copies due to IR

^c^Gene that contains two introns

^d^*ycf*1 spans an inverted repeat region (IRa) and the adjacent small single-copy region (SSC). *ycf1*-like is a truncated form of *ycf1 *that occurs in the inverted repeat region IRb.

^e^Encodes a putative protein similar to *ycf15 *from *Lactuca sativa *(ABD47292.1) and *Helianthus annuus *(ABD47205.1)

^f^Gene that undergoes trans-splicing

The border sequences between the IR, LSC and SSC regions vary among different species. The pattern in melon is similar to that in cucumber, as described in [45]. In particular, IRa extended 1,199 bp into the *ycf1 *gene, and the IRb/SSC border was within the coding regions of the *ycf1*-like and *ndhF *genes, which overlap by 32 bp. The IRa/LSC border was located downstream of the *trnH*-GUG gene, whereas the ψ*rps19 *gene, present in that region in other species such as *Arabidopsis thaliana*, was absent in both melon and cucumber. Finally, the IRb/SSC border extended 2 bp into the 5' coding region of the *rps19 *gene, as in cucumber.

The melon chloroplast genome was screened for simple sequence repeats (SSRs), which resulted in the identification of 69 microsatellites that were at least 10 nt in length (1 to 2 nt repeats) or contained at least four tandem repeat units (3 to 6 nt repeats). All the microsatellites found were shorter than 18 bp. SSRs accounted for 796 bp (0.5%) of the total sequence, which was similar to the SSR content estimated for the melon nuclear genome [48]. The poly(A)/poly(T) microsatellite was the only mononucleotide repeat found and represented 79.7% of all SSRs found.

### Comparison of the cucumber and melon chloroplast genomes

As of today, the chloroplast genome of only one cucurbit species, *Cucumis sativus *(cucumber), has been published [45-47]. Previous studies have suggested that sequence analysis of chloroplast genes can be a valuable tool for phylogenetic studies among closely related species [49,50]. Accordingly, and due to the highly polymorphic nature of the *Cucurbitaceae *family, a comparison of the melon and cucumber chloroplast genomes can provide useful information about the evolutionary relationships among cucurbit species.

The chloroplast genome sequence of the *C. sativus *'Chipper' line (GenBank Acc. No. DQ865976.1) was compared to the melon genomic sequence reported here. The cucumber genome was 494 bp shorter than the melon genome, but overall only approximately 5% of the nucleotide sequences were different, mainly due to indels and SNPs (Table 3). Deletions in the melon sequence, compared to cucumber, were found at 237 loci and represented 2,742 bp, or 1.76% of the cucumber genome. Eighty-five percent of the deletions involved the loss of less than 10 bp, while five deletions represented the loss of 125 to 379 bp. Insertions in the melon sequence as compared to cucumber were found at 188 loci representing 3,210 bp. Seventy-one percent of these insertions involved the gain of less than 10 bp; six insertions of 126 to 714 bp were also found. Additionally, we identified 2,250 SNPs, which represented 1.44% of the melon sequence.

**Table 3:** Differences between the *C. melo *and *C. sativus *chloroplast genome sequences^a^

**Deletions**^1^						
	length (bp)		number			
	1		88			
	2		21			
	3-4		26			
	5-6		54			
	7-9		14			
	10-19		14			
	22-84		11			
	125-270		2			
	353-379		3			
Total:	**2742 **(1.76%^2^)		237			
**Insertions**^3^						
	**length (bp)**		**number**			
	1		76			
	2-3		17			
	4-5		18			
	7-8		11			
	9		11			
	10-17		8			
	18		6			
	19-87		12			
	126		2			
	147		2			
	714		2			
**Total:**	**3210 **(2.06%^4^)		**188**			

**SNPs**^5^						
	**C→A** **G→T**	**} **507	**C→T** **G→A**	**} **437	**A→G** **T→C**	**} **420
	**A→C** **T→G**	**} **392	**C→G** **G→C**	**} **254	**A→T** **T→A**	**} **240
**Total:**	**2250 **(1.44%)					

**Other polymorphisms**^5^						
			**number**		**length**	
	**GA→TT** **GTGG→AATC** **CCAT→TTTA** **TTAT→AATC**		1 1 1 1			
	**Highly polymorphic regions**^6^		8		709 bp	

^a^The chloroplast genome sequence of *Cucumis sativus *'Chipper' line (GenBank Acc. No. DQ865976.1) was used for the comparison

^1^Positions where the *C. melo *sequence has a gap in comparison to cucumber

^2^Relative to the cucumber genome length

^3^Positions where the *C. sativus *sequence has a gap in comparison to melon

^4^Relative to the melon genome length

^5^Cucumber → melon

^6^Highly divergent regions found between the melons at coordinates 126,000 and 130,000

Recombination mechanisms between direct repeat sequences on the SSC/IR border regions have been found to be responsible for the expansion/contraction of the IR sequences, which can create large sequence variations in chloroplast genomes [45,51]. Significantly, the area of highest diversity between the compared genomes was found in the region located between the melon sequence coordinates 126,000 and 130,000, close to the SSC/IRa border. In particular, eight highly polymorphic regions with a total length of 709 bp were found in this region (Table 3).

An additional comparison between the amino acid sequences of the melon and the cucumber chloroplast-encoded proteins was performed, and the results are shown in Additional file 1 Table S1. Except for ORF70 and the *ycf1*-like gene the annotation of both species contained the same set of ORFs. Nevertheless, the published cucumber sequence contains ORFs homologous to those of melon ORF70 and *ycf1*.

When the predicted protein sequences were BLASTed against the non-redundant GenBank database, cucumber was identified as the highest-scoring plant species for 72 of the 81 predicted coding genes (duplicated genes were counted as one gene). With the exception of the *rpl22 *and *accD *genes, which had identity values of 91% and 82%, respectively, the rest of the 72 genes showed identity values higher than 95% when compared to their cucumber homologues.

Five out of nine genes whose highest-scoring match was not cucumber showed protein identities higher than 96%, although the identity values, when compared to their cucumber homologues, were also high (Additional file 1 Table S1). Finally, the predicted proteins with lower identity to other plant chloroplast proteins were those encoded by the *clpP*, *ycf2 *and, particularly, both the *ycf1 *and *ycf1*-like genes.

### Organisation of the *Cucumis melo *mitochondrial genome

After the isolation of intact mitochondrial organelles from young melon leaves, mtDNA was extracted and sequenced using the Roche-454 technology, and the 104,462 resulting reads were assembled as described in the Methods section. BES from two different BAC libraries [13] and whole genome sequences derived from 454 sequencing of 3-kb, 8-kb and 20-kb paired-end (PE) libraries (unpublished) were also used to improve the genome assembly.

The resulting sequence amounts to 2.74 Mb distributed in five scaffolds of lengths 2,428,112 bp, 147,837 bp, 107,070 bp, 47,488 bp and 6,086 bp and four additional unscaffolded contigs that totalled 1,809 bp. (Table 4). The overall sequence coverage is 18-fold. The size of the melon mitochondrial genome has previously been estimated to be approximately 2.4 to 2.9 Mb [27,30]. Based on this estimate, we can assume that 95% of the mitochondrial genome has been assembled and that 84% of the genome is contained in a single scaffold. Failure to assemble all the reads in a single circular sequence can be attributed to the high degree of repetitive sequences found in this genome, as will be discussed later. However, the existence of several subgenomic molecules that coexist inside the mitochondria, as has been described in other species [52-54], cannot be ruled out. The contig and scaffold sequences have been deposited in GenBank under Accession Numbers JF412792 to JF412800.

**Table 4:** *C. melo *mitochondrial genome characteristics

**Total scaffold/contig size [nt]**	2,738,402
**GC content**	44.5%
**Gene number^a^**	78
**Protein genes^a^**	51
**rRNA genes^a^**	3
**tRNA genes^a^**	24
**Genes with introns**	10
**Trans-spliced genes**	3
**Coding sequence**	1.68%
**Protein coding**	1.37%
**tRNAs and rRNAs**	0.31%
**Non-coding sequence**	98.32%
***cis*-spliced introns**	1.80%
**Intergenic sequences**	96.53%
**Repetitive content**	
**SSRs**	0.15%
**Transposable-related sequences**	0.24%
**Any perfect repeats**	42.70%
**Tandem repeats**	1.51%
**Inverted repeats**	1.85%
**Mitochondrial-like^b^**	4.4%
**Chloroplast-like^c^**	1.41%
**Nuclear-like^d^**	46.47%

^a^Duplicated and triplicated genes (see Table 5) were counted once

^b^Homologous regions between *C. melo*, *C. lanatus *and *C. pepo *mitochondrial genomes

^c^Homologous regions between *C. melo *mitochondrial and chloroplast genomes

^d^Homologous regions between *C. melo *nuclear and mitochondrial genomes

The GC content of the mitochondrial genome was found to be 44.5%, which is higher than that of the chloroplast and nuclear melon genomes and similar to the estimated GC content of the watermelon and squash mitochondrial genomes [27]. Annotation of the sequence was performed, and 67 genes were detected (Tables 4 and 5). Gene-coding regions accounted for 1.7% of the genome (45,926 bp) and included 36 protein-coding genes and 4 conserved ORFs, 3 rRNA genes and 24 tRNA genes, which represented 1.3%, 0.1% and 0.3% of the total sequence, respectively; *cis*-spliced introns accounted for 1.8% of the genome. The genes *nad2*, *cox1*, *ccmFc*, *rpl2*, *rps3 *and *rps10 *contained one intron, *nad4 *contained three introns, and *nad1*, *nad5 *and *nad7 *contained four introns each. The *nad*1, *nad*2 and *nad*5 genes were found to undergo trans-splicing.

**Table 5:** List of genes found in *Cucumis melo *mitochondrial genome

**RNA genes**					
**tRNAs**	*trnD*-GTC^a^	*trnE*-TTC^b^	*trnF*-GAA	*trnfM*-CAT^b^	*trnG*-GCC^b^
	*trnH*-GTG^b^	*trnH*-GTG-cp^c^	*trnI*-CAT^b, d^	*trnL*-CAA^b^	*trnM*-CAT
	*trnM*-CAT-cp^c^	*trnN*-GTT	*trnN*-GTT-cp^c^	*trnP*-TGG	*trnQ*-TTG
	*trnR*-ACG	*trnR*-ACG-cp^c^	*trnS*-GCT^b^	*trnS*-TGA	*trnS*-TGA-cp^c^
	*trnW*-CCA^e^	*trnY*-GTA	Ψ*trn^f^*	Ψ*trnC*	
**rRNAs**	*rrn26*	*rrn18*	*rrn5*^a^		
**Complex I** **(NADH dehydrogenase)**	*nad1*^g, h, i^	*nad2*^i, j^	*nad3*	*nad4*^k^	*nad4L*^g^
	*nad5*^h, i^	*nad6*	*nad7*^h^	*nad9*	
**Complex II** **(succinate dehydrogenase)**	*sdh3*	*sdh*4			
**Complex III** **(ubiquinol cytochrome c reductase)**	*cob*				
**Complex IV** **(cytochrome c oxidase)**	*cox1*^j^	*cox2*	*cox3*		
**ATP synthase**	*atp1*	*atp4*	*atp6*	*atp8*	*atp9*
**Other genes**					
**Cytochrome C biogenesis**	*ccmB*	*ccmC*	*ccmFc^j^*	*ccmFn*	
**Transport membrane**	*mttB*				
**Maturase**	*matR*^l^				
**Ribosomal proteins**	*rpl2*^j^	*rpl5*	*rpl16*^m^	*rps1*	*rps3*^j^
	*rps4*	*rps7*	*rps10*^g, j^	*rps12*	*rps13*
**Conserved ORFs**	ORF1^n^	ORF2^o^	ORF3^p^	ORF4^q^	

Pseudogenes are symbolised by ψ

^a^Three gene copies

^b^Two gene copies

^c^Chloroplast origin

^d^C assumed to be post-transcriptionally modified to lysidine, which pairs with A, not G (see PubMed ID 1698276)

^e^Seven gene copies

^f^Undetermined anticodon

^g^RNA editing creates a codon

^h^Gene contains five exons

^i^Gene undergoes trans-splicing

^j^Gene contains one intron

^k^Gene contains four exons

^l^Start codon not determined

^m^Alternative start codon (see PubMed IDs 8193306 and 9327595)

^n^Similar to chloroplast *ycf2 *gene

^o^Similar to ORF150 in *V. vinifera*, ORF159b in *Nicotiana*, ORF168 in *Marchantia *and ORF187 in *Physcomitrella*

^p^Similar to amino acid sequence GenBank ID CAA69750.1

^q^Similar to 5' fragment of photosystem I P700 apoprotein A1

As of 6 June 2011, the mitochondrial genome sequences of 32 Streptophyta have been deposited in GenBank [25], including two cucurbit species: *C. lanatus *(NC_014043) and *C. pepo *(NC_014050). Genes homologous to all the predicted protein-coding genes from the watermelon and squash mitochondrial genomes have been found in the annotated melon sequence, with the exception of the *rps19 *gene. However, it is already known that this gene has been lost from the mitochondrial genome in diverse species due to transfer to the nucleus; in particular, cucumber, which is phylogenetically closer to melon than both watermelon and squash, has apparently recently lost this gene [55]. Apart from the loss of the *rps19 *gene, some differences were found regarding the number of tRNA genes in the three cucurbit genomes. For example, while two *trnQ *genes, two *trnC *genes and one *trnK *gene were found in watermelon, only one *trnQ *gene and no *trnC *or *trnK *genes were present in melon. However, it is well known that even phylogenetically related species differ substantially in their tRNA complement set (for example, see [30] for cucurbits).

When the predicted protein sequences were BLASTed against the non-redundant GenBank database, cucurbits were identified as the highest-scoring plant species in only 16 of all 40 predicted coding genes (Additional file 2 Table S2). This is in sharp contrast to the chloroplast sequences discussed above, in which the majority of melon proteins displayed the highest identity values when compared to their cucumber homologues. The identity values of the 16 proteins ranged from 78% (rps3 protein) to 99% (cob protein). However, RNA editing events, which are known to frequently alter mitochondrial transcripts, have not been identified in melon except for a limited number of cases. Therefore, the actual identity values are expected to be somewhat higher than our estimated values. Twenty out of 24 genes whose highest-scoring match was not a cucurbit species showed protein identities higher than 90% for the corresponding best hits. Finally, the predicted proteins with lower identity to other plant mitochondrial proteins are those encoded by *sdh3*, *ccmFn*, *rps1*, *rps4 *and, particularly, ORF2, ORF3 and *rps3*.

For gene distribution along the mitochondrial chromosome, several small syntenic clusters are found when the melon, watermelon and squash mitochondrial sequences are compared (Additional file 3 Figure S1). However, as has been described for watermelon and squash, the distribution of these clusters reveals a high level of genomic shuffling and rearrangement between these three species [30].

### Analysis of repetitive DNA, chloroplast and nuclear-derived DNA

**Figure 2:**
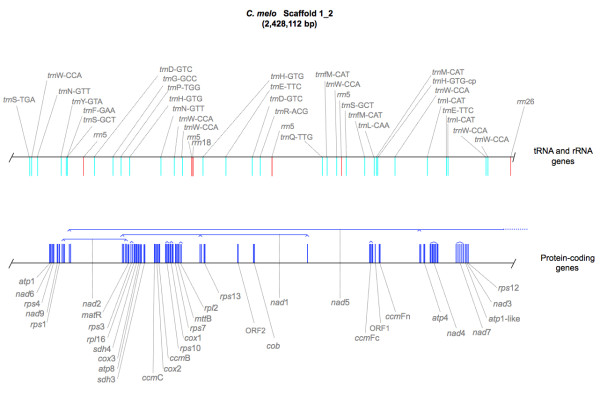
**Gene density representation of 2.43 Mb of the melon mitochondrial genome**. The displayed region corresponds to the largest scaffold obtained, which represents 84% of the estimated melon mitochondrial genome. The symbol ^ connects exons of the same gene, while horizontal lines connect exons of trans-spliced genes. The nad5 gene contains five exons, of which only four are present in the depicted scaffold.

Although the gene content of melon is highly similar to that of watermelon or squash, the melon mitochondrial genome size is thrice that of squash and as much as seven times that of watermelon. In fact, regions of DNA as large as 600 kb could be found that contained no protein-coding genes. Figure 2 shows a schematic representation of the gene density of the largest scaffold (2.43 Mb).

To establish the fraction of this huge genome that is shared with the other two cucurbit mitochondrial genomes, all three sequences were cross-compared using BLASTn. It has been previously reported that processes such as nuclear or chloroplast DNA transfer to the mitochondria and internal recombination of the mitochondrial genome lead to a high degree of sequence rearrangement that can obscure any trace of homology over time [30]. For this reason, a less conservative e-value of 1E-3 was chosen for the comparative analysis. As a result, 173 kb (46%) and 163 kb (16.6%) of the watermelon and squash mitochondrial genomes, respectively, were found to be homologous with the melon sequence. In addition, 73% of these homologous regions (119 kb from watermelon and 125 kb from squash) were shared among all three species. Seventy-nine regions longer than 500 bp accounted for 60% of the total homologous sequence (1,000 homology regions averaging 180 bp in length). These figures are in accordance with the generally accepted theory of watermelon being phylogenetically closer to melon than to squash.

The conserved mitochondrial-like sequence was found to contain all the predicted ORFs except for ORF1 and ORF4 (which are present in conserved regions in melon and watermelon, but not squash), and so it can be concluded that the approximately 120 kb of conserved sequence (32%, 12% and 4.4% of the watermelon, squash and melon mitochondrial genomes, respectively) represented a core cucurbit mitochondrial genome present in all three sequenced genomes. Also, the finding that approximately 27% of the conserved melon and watermelon regions were not conserved in squash, and *vice versa*, points to independent events that have directed the evolution of these three genomes from a common cucurbit ancestor. In any case, the previous data showed that 95% of the melon mitochondrial genome had no homology whatsoever with the mitochondrial sequences of other cucurbits.

Previous reports have indicated that small, repetitive DNAs contribute significantly to the expanded mitochondrial genome of cucumber, which is estimated to be 1.8 Mb [31]. Therefore, the presence of SSRs, transposable elements, inverted repeat regions and tandem and direct repeats was analysed. The mitochondrial sequence contains 357 SSRs (one SSR every 7.7 kb) that amounts to 4,071 bp (0.1% of the total sequence). All the microsatellites were shorter than 21 bp, except for a (GACT)_7_. This value is ten times lower than the estimated SSR content of the melon nuclear genome [48]. In comparison, the squash and watermelon mitochondrial genomes contain one SSR every 4.6 kb and 5.6 kb, respectively (0.3% and 0.2% of the total sequence). Therefore, microsatellites represent an insignificant portion of the melon mitochondrial genome and cannot explain its large size. The presence of transposon-related sequences was also investigated, but only small fragments that totalled 6,480 bp (0.23% of the total sequence) were found to show homology to transposable elements (mainly LTR retrotransposons). The search for inverted repeat sequences (IRs) produced 427 pairs of IRs, which amounted to 50,601 bp (1.8% of the available mitochondrial sequence). Percent matches between IRs were higher than 70%, with 137 pairs of IRs showing values higher than 95%. The average repeat length was 82 bp; the longest IR found was 1,067 bp. In comparison, the IR contents of watermelon and squash were also calculated, but only 14 IRs (1,497 bp) and 17 IRs (2,096 bp) were found in those species, which is between four and nine times lower than the melon IR content. Therefore, the melon mitochondrial genome was significantly enriched in sequences that can mediate recombination events.

Regarding the tandem repeat content of the sequenced genome, the analyzed sequence contained 449 tandem repeats, which amounted to 41,212 bp or 1.5% of the available sequence. The average period size and period copy number were 39 and 3, respectively. The most abundant type of tandem repeats were those with period sizes of 29, 35 and 70, which totalled 40% of all tandem repeats found and 56% of the tandem distributed sequence. As a comparison, the tandem repeat contents of watermelon and squash were also calculated, and 10 repeats (1,036 bp or 0.3% of the genome) and 236 repeats (19,060 bp or 1.9% of the genome), respectively, were found. Therefore, while the relative tandem repeat contents of melon and squash were similar, watermelon showed a significantly reduced tandem repeat content in its mitochondrial genome.

Additionally, the maximal repeat content and repeat families of the mitochondrial genomes of watermelon, squash and melon were calculated using two different programs (see Methods section). The RepeatScout program, which detects repeats larger than 50 bp and excludes low complexity sequences, predicted 101 repeat families (with an average copy number per family of 35) in melon, 13 families (with an average copy number per family of 34) in squash and 2 families (with an average copy number per family of 3) in watermelon. The most abundant repeat families for the compared mitochondria consisted of 365 copies of approximately 120-bp-long repeats for melon and 90 copies of approximately 173-bp-long repeats for squash. Only 3 repeats were found to be longer than the average read length, which is 399 nt. Incidentally, the fact that the most abundant repetitions are shorter than the average 454 read length implies that, in those cases, the 454 reads extends the repetitions and result in the correct assembly of reads. Therefore, although the existence of mis-assemblies of repetitive sequences cannot be completely rule out, mis-assemblies probably affect our proposed sequence to a much lower degree that could be guessed based only on the high repeat content of the genome.

We also searched for exact repeats longer than 20 bp using REPuter (results summarised in Table 6). Similar to the findings reported for squash [30], we found a significant content of short repeats in the mitochondrion of melon. Our numbers for squash and watermelon were slightly lower compared to data obtained in [30] because we looked only for exact repeats, but the differences among the genomes analysed are clear. The mitochondrion of melon is much richer in large repeats than that of squash.

**Table 6:** Repeat content in the mitochondria of *Cucumis melo*, *Cucurbita Pepo *and *Citrullus lanatus*

	Repeat coverage (%)		
Repeat length (# nt)	*C. melo*	*C. pepo*	*C. lanatus*
20-29	17.16	15.33	1.65
30-39	7.12	4.30	0.57
40-49	3.75	1.60	0.35
> 50	14.67	4.15	5.76
All	42.70	25.39	8.33

Chloroplast-derived DNA accounts for as much as 9% of sequenced plant mitochondrial genomes [56]. The melon chloroplast genome described above was used to identify mitochondrial sequences of putative chloroplast origin. In all, 35 mitochondrial regions that ranged from 61 to 10,578 bp (average 1.1 kb) and totalled 38.6 kb or 1.4% of the mitochondrial genome, showed homology with the melon chloroplast sequence. On the other hand, 54 kb or 35% of the melon chloroplast genome showed homology to the mitochondrial genome. The 38.6 kb difference in the chloroplast-derived mitochondrial sequence was due to duplicated regions in the chloroplast genome. As a comparison, watermelon's mitochondrion contains 23 kb of chloroplast-like sequences, while squash's mitochondrion contains 113 kb, which represents approximately 80% of other sequenced cucurbit chloroplast genomes such as those of melon and cucumber. Therefore, no correlation seems to exist between the mitochondrial sizes of these three species and their chloroplast-derived sequence content.

Finally, nuclear-derived sequences have been detected in several plant mitochondrial genomes and amount to up to 7% of their size [30,57]. In watermelon and squash, approximately 20 kb of nuclear-like sequences, most of which resemble retrotransposable elements, have been found. Although the contribution of retrotransposons to the expanded melon mitochondrial genome is negligible, as discussed above, the BLASTing of 361 Mb of the melon nuclear genome draft sequence obtained in our laboratories (unpublished data) against the mitochondrial sequence produced 1,114 mitochondrial regions that ranged from 193 bp to 10,355 bp and that totalled 1,272,615 bp (46.5% of the available mitochondrial sequence). Significantly, even when only the 413 homologous fragments longer than 1 kb were considered, more than 33% of the available mitochondrial sequence still showed homology with melon nuclear regions. The analysis of those 37 mitochondrial homologous regions longer than 4 kb and totalling *ca*. 200 kb showed that the average identity between the mitochondrial and nuclear regions was 91% with values ranging from 84 to 96%. The detailed analysis of two of these regions with lengths 4,220 and 4,044 bp and identities of 94% and 89% relative to their nuclear counterparts, showed a transition/transversion mutation ratio of 2.2 and 3.8 respectively, with C_Mit _→ T_Nuc _and G_Mit _→ A_Nuc _the most abundant mutations found, and C_Mit _→ A_Nuc _the most representative transversion mutations. Twenty-seven indels totalling 70 nt and five gaps of between 11 and 60 nt were also found.

Interestingly, all 37 regions analyzed but three displayed high levels of sequence identity with at least two different nuclear regions, therefore suggesting a relationship between the repetitive nuclear DNA and the mitochondrial DNA of putative nuclear origin.

A large fraction of the mitochondrial gene-containing regions and some chloroplast-like regions in the mitochondria showed homology with the nuclear sequence, as was expected because many mitochondrial genes have homologous counterparts in the nuclear genome. Also, DNA transfer from the chloroplast to the mitochondrion has been known to occur. When these regions were not considered, 1.14 Mb of mitochondrial sequence still showed homology to nuclear sequences. In all, nearly half of the melon mitochondrial genome seemed to be of nuclear origin; therefore, the transfer of DNA from the nucleus can, at least partially, explain the large size of this mitochondrial genome. However, the nature of approximately 1.5 Mb of mitochondrial sequence remains to be elucidated.

## Conclusions

Whereas the size and gene organisation of the chloroplast genome were similar among cucurbit species, the mitochondrial genomes showed a wide variety of sizes, with a non-conserved structure both in gene number and organisation, as well as in the features of the noncoding DNA; nevertheless, we identified a minimum cucurbit genome core of 119 kb between melon, watermelon and squash with a high level of nucleotide sequence conservation. In addition to a high proportion of repetitive DNA content in melon, compared to watermelon and squash, the transfer of nuclear DNA to the melon mitochondrial genome seems to explain the size of the largest mitochondrial genome reported so far.

## Methods

### Source of the chloroplast genome sequences

A melon random-shear BAC library had been previously constructed and the BES from 16,128 clones determined [13]. The average sequence length was 534 bp. The BES were then filtered using the cucumber chloroplast genome sequence (GenBank Acc. No. DQ865976.1) as a reference, and 5,785 BES totalling 3.2 Mb were found to show homology with the cucumber sequence.

### Chloroplast genome assembly, annotation and analysis

The selected BES were assembled using the Sequencher 4.1.1 software package with a minimum overlap of 15 and a minimum match of 85%. Due to the presence of an inverted repeat in the chloroplast genome of plant species, a final step of manual assembly was required to obtain a final contig of 5,683 sequences that represented the melon chloroplast genome.

The consensus sequence was then annotated using the DOGMA online organellar annotation tool [58]. The predicted ORFs, including *cis*- and *trans*-splicing sites, were manually checked by comparison with all other published chloroplast genes, and several changes were then introduced into the DOGMA preliminary annotation to produce the final annotated sequence. A graphical representation of the annotated genome was produced using the CGViewer Server [59].

The melon and cucumber chloroplast genome sequences were aligned using MEGA4 software to detect polymorphisms between these species. The predicted chloroplast-encoded proteins were analysed for homology with other known proteins using the GenBank non-redundant protein database and the BLASTP software. Microsatellites were searched using msatcommander 0.8.2 software [60]. SSRs considered for the final dataset included 1- to 2-nt repeats of at least 10 nt in length and 3- to 6-nt repeats with at least four unit repetitions.

### Plant material

Melon seeds from the double haploid line PIT92 (derived from the cross PI 161375 × T111) were germinated inside a Petri dish in a dark growth chamber for 3 days at 25°C. After germination, the seeds were planted in pots that contained synthetic soil and maintained in a greenhouse at 26 ± 2°C and with day/night cycles of 16/8 h, respectively. The PIT92 melon line was also used for construction of BAC libraries [12,13] and has been used for whole genome sequencing (Garcia-Mas *et al*., unpublished).

### Isolation of mitochondrial DNA from intact mitochondria

Intact mitochondrial organelles were isolated from young melon leaves according to a modification of a previously described method [61]. Fifty grams of young melon leaves were manually harvested, cut into 10- to 20-mm lengths and ground in a Polytron PT2000 homogeniser with 120 ml of grinding medium at 4°C. The homogenate was filtered through four layers of Miracloth, placed into 6 × 50 ml Nalgene tubes and centrifuged for 5 min at 3,200 rpm with a JA14 rotor in a Beckman Coulter centrifuge (Avanti J-26 XP). The supernatant was then re-centrifuged for 20 min at 13,600 rpm, and the resulting pellet was resuspended in 5 to 10 ml of 1× wash buffer, transferred to a 50 ml Nalgene tube and centrifuged for 5 min at 3,200 rpm with a JA17 rotor. After centrifugation, the supernatant was transferred to a new tube and re-centrifuged at 13,600 rpm for 20 min. The resulting pellet was thoroughly dispersed with a fine paintbrush in 5 ml of washing buffer, layered over a 0 to 5% PVP gradient made earlier and centrifuged for 40 min at 21,000 rpm in a Beckman Coulter ultracentrifuge (Optima L-90 K). After centrifugation, the mitochondria formed a white-yellow colour band toward the bottom of the gradient, which was carefully recovered with a syringe, transferred to a new 50-ml Nalgene tube with 1× wash buffer and concentrated in a pellet with 3 wash centrifugation steps at 13,600 rpm for 15 min. After organelle isolation, mitochondrial DNA was lysed and purified as described [62].

### Mitochondrial genome sequencing and assembly

Sequencing was performed using the Roche Genome Sequencer FLX System on 1/8 of a Titanium Microtitre Plate. The filtering process was passed by 120,802 sequences, which contained 48,154,028 bases with an average length of 399 nt. Duplicate reads were identified using the cd-hit-454 program [63], and 104,462 nonredundant reads were assembled using Newbler (version 2.5 beta) to produce a set of contigs totalling 2.711 Mb. The obtained contigs (except for the 64 contigs out of 539 that had < 10× coverage) were used as a query for BLASTing [64] against additional pools of sequences (obtained from the same genotype, PIT92, used in this study) that were available in our laboratory: BES from two different BAC libraries [13] and whole genome sequences derived from Roche 454 sequencing of 3-kb, 8-kb and 20-kb paired-end libraries (unpublished). Raw BESs were filtered and trimmed for quality and vector contamination using SeqTrim [65]. Only BESs that had > 98% identity to the query for over 80% of their length were considered. In cases in which the BESs were paired (when both 5' and 3' ends of the same BAC insert were available), both pairs were taken if only one pair met the described conditions. At the end, there were 1,822 BESs (97.5% paired) used in this study. For the 454 whole genome PEs, we created a database from a subset of nonredundant and "true" PEs (sequences that contained the 454 linker flanked on each side by > 50 nt of sequence). We retrieved sequences that had > 99% identity to the query and ended up with 10,724 3-kb, 14,723 8-kb and 2,683 20-kb PEs.

Assemblies were performed using two different programs: Newbler (version 2.5 beta) and MIRA (version 2). Newbler is able to sort contigs into scaffolds using the PEs but is often unable to incorporate conserved repeats into these scaffolds, which leaves gaps of approximated sizes based on paired-end insert distances (repeats are often assembled into "collapsed" contigs that remain orphaned after the assembly). In contrast, MIRA is unable to build scaffolds, but it tries to differentiate copies of conserved repeats and include them with the rest of non-repeat contigs. Therefore, contigs derived from MIRA were used, when possible, to close the gaps in the scaffolds obtained with Newbler or to join two or more scaffolds. A detailed summary with the metrics of the assembly process can be found in the Additional file 4 Table S3.

### Mitochondrial genome annotation and analysis

A nucleotide database was built that contained the predicted cDNAs from all the sequenced Streptophyta mitochondrial genomes, as previously published [25]. BLASTN searches were performed, and each individual ORF found was checked by comparison with all other mitochondrial proteins published; several changes were then introduced to produce the final annotated sequence. Structural RNA genes were identified using tRNAscan-SE 1.21 (for tRNAs) and RNAmmer 1.2 (for rRNAs) software [66,67].

The predicted mitochondrially encoded proteins were analysed for homology with other known proteins using the GenBank non-redundant protein database and the BLASTP software. Microsatellites were searched using the msatcommander 0.8.2 software. SSRs considered for the final dataset included 1- to 2-nt repeats of at least a 10 nt length and 3- to 6-nt repeats with at least four unit repetitions. Transposable-related sequences were identified using CENSOR online (with default sensitivity parameters and *Arabidopsis thaliana *as a reference DNA source) [68]. Tandem repeats were analysed using the Tandem Repeats Finder software [69] (min. align. score 60; max. period size 2,000). Inverted repeats were detected using the Inverted Repeats Finder software [70] (match 2; mismatch 3; delta 5; match probability 80; indel probability 10; Minscore 40; Maxlength to report 500,000; MaxLoop 500,000). Two different programs were used to look for duplicated DNAs and to repeat family classification in the sequences of interest: REPuter and RepeatScout, respectively, with default parameters [71,72]. Results from REPuter were analysed to avoid overestimating the total repeat content due to repeat overlaps.

Nuclear-like mitochondrial regions were identified by performing a BLASTN with e-value < 1E-100 (corresponding approximately to a hit of 200 nt and 90% identity or a 400 nt hit with 85% identity) against a melon nuclear genome draft that has been produced in our laboratories [Garcia-Mas *et al*., manuscript in preparation]. Chloroplast-like regions were identified by performing a BLASTN analysis with e-value < 1E-40 against the assembled melon chloroplast genome reported in this paper.

Comparisons to the *C. lanatus *and *C. pepo *mitochondrial genomes (GenBank Acc. Nos. GQ856147 and GQ856148) were performed using BLASTN with e-values < 1E-3.

## Authors' contributions

LRM performed isolation of mitochondria and purification of mitochondrial DNA. VMG conducted the assembly, annotation and analysis of the chloroplast genome. AB carried out the assembly of the mitochondrial genome and provided bioinformatic analysis support. LRM and VMG conducted the annotation and analysis of the mitochondrial genome and helped completing the manuscript. MCM taught LRM how to isolate mitochondria and participated in the isolation. MAA generated and sent to LUCIGEN^® ^plant melon material for the construction of the random shear BAC library. PP is the main coordinator of the MELONOMICS project and participated in the conception of the study together with MAA and JGM. JGM is the principal investigator and coordinated the writing of the manuscript. All authors read and approved the final manuscript.

## Supplementary Material

Additional file 1 **Table S1**. Protein homologies between *C. melo *and other plant chloroplast genomes. Click here for file

Additional file 2 **Table S2**. Protein homologies between *C. melo *and other plant mitochondrial genomes. Click here for file

Additional file 3 **Figure S1**. Syntenic relationships between the mitochondrial genomes of *Cucumis melo*, *Citrullus lanatus *and *Cucurbita pepo*. Only the protein coding regions have been used for this analysis. Intronless genes are depicted as orange vertical lines. Individual colours are used for the exons of each gene with introns. Click here for file

Additional file 4 **Table S3**. Metrics of the *Cucumis melo *mitondrial genome assembly. Click here for file
