# Supplementary figures and images for: Determination of the melon chloroplast and mitochondrial genome sequences reveals that the largest reported mitochondrial genome in plants contains a significant amount of DNA having a nuclear origin
Source: BMC Genomics. 2011 Aug 20;12:424. doi: 10.1186/1471-2164-12-424 (PMC3175227; doi:10.1186/1471-2164-12-424)

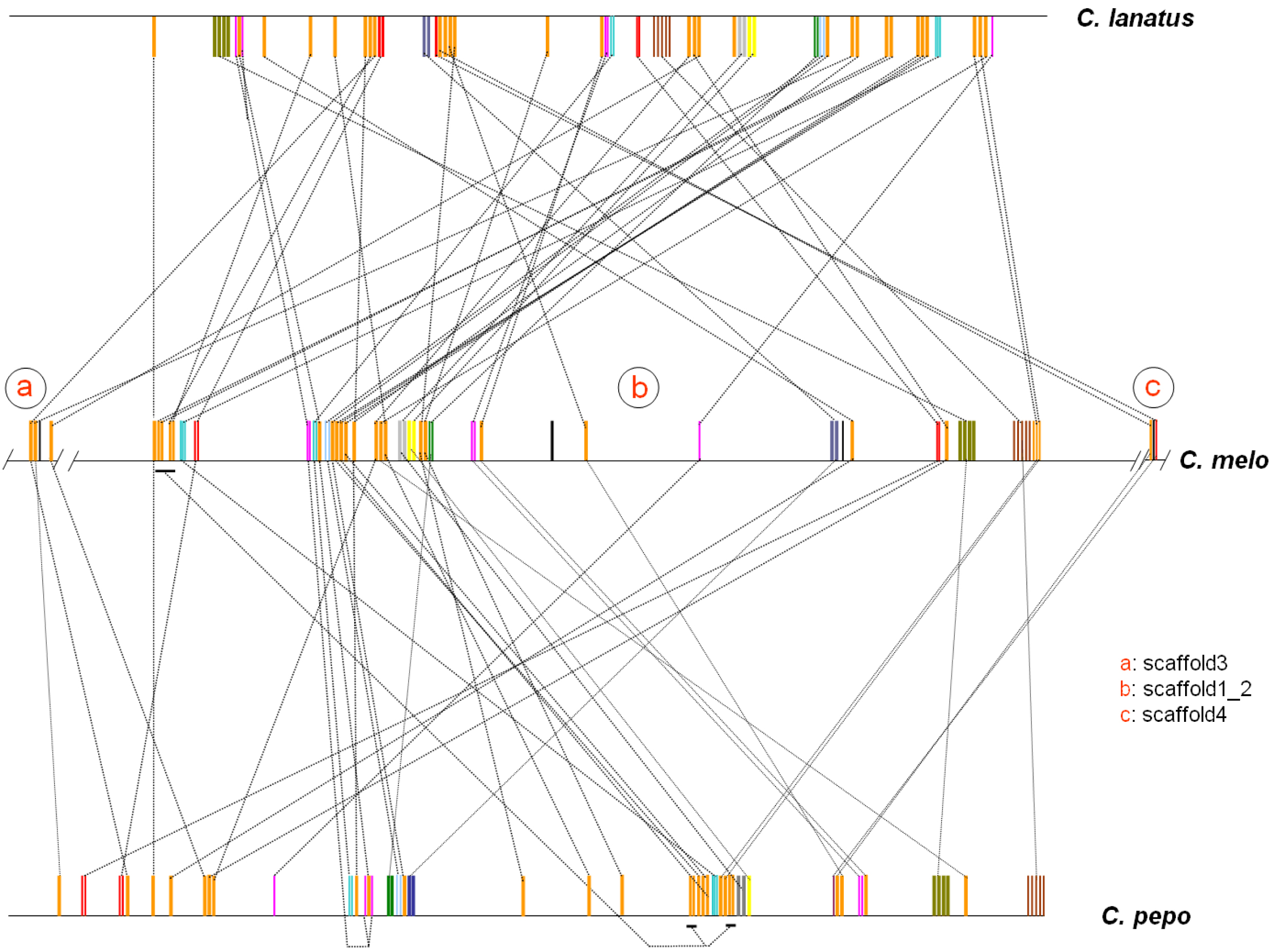

Supplement: Additional file 3 — Figure S1. Syntenic relationships between the mitochondrial genomes of Cucumis melo, Citrullus lanatus and Cucurbita pepo. Only the protein coding regions have been used for this analysis. Intronless genes are depicted as orange vertical lines. Individual colours are used for the exons of each gene with introns. [file 1471-2164-12-424-S3.JPEG]
